# Knowledge and Attitudes Toward Organ Donation Among Students and Professionals in Medical Colleges in the Eastern Part of India

**DOI:** 10.7759/cureus.70556

**Published:** 2024-09-30

**Authors:** Ekta Krishna, Shibajee Debbarma, Alok Ranjan, Sanjay Pandey, Vijay Kumar, Vijay Kumar, Madhusudan P Singh, Akanksha Yadav

**Affiliations:** 1 Community and Family Medicine, All India Institute of Medical Sciences, Patna, IND; 2 Radiodiagnosis, Indira Gandhi Institute of Medical Sciences, Patna, IND; 3 Community Medicine, Indira Gandhi Institute of Medical Sciences, Patna, IND; 4 Pharmacology, All India Institute of Medical Sciences, Raipur, IND

**Keywords:** attitude, awareness, knowledge, medical professionals, medical students, organ donation

## Abstract

Background: Organ transplantation is a critical, life-saving procedure revolutionizing healthcare. Despite medical advancements, there remains a global shortage of organs available for transplantation. This is more pronounced in India where the organ donation rate is significantly low.

Objective: To assess the knowledge and attitude toward organ donation among students and professionals in medical colleges of Patna, Bihar.

Methods: A cross-sectional study was conducted in three government medical colleges located in Patna that included 666 participants comprising faculty, resident doctors, nursing staff, medical and nursing students, and interns. Data were collected using a pre-designed structured questionnaire comprising a socio-demographic profile and knowledge and attitude of participants toward organ donation. The data were analyzed using descriptive statistics and multivariate analysis.

Results: The mean age of study participants was 23±4.24 (SD). The majority (504, 75.7%) of participants were aged between 21 and 30 years and the majority (372, 55.9%) were females. There were significant differences in knowledge about organ donation across groups. Faculty and resident doctors demonstrated higher awareness (113, 73.9%) compared to others. Attitude toward organ donation was positive overall, with the majority (614, 92.2%) supporting organ donation campaigns. However, misconceptions about organ donation and its legal aspects persisted among participants.

Conclusion: There is a need for targeted educational intervention to improve knowledge and dispel myths about organ donation among medical students and professionals.

## Introduction

Organ transplantation is a life-saving medical procedure that has revolutionized healthcare, offering hope to patients suffering from organ failure [1]. Despite significant advancements in medical science, the shortage of organs for transplantation remains a critical issue globally [2]. This disparity is evident in India, where the gap between demand and availability of organs for transplantation is widening, leading to a high mortality rate among patients in need. The Ministry of Health and Family Welfare, Government of India has estimated a shortage of approximately 1,75,000 kidneys, 50,000 livers, hearts, and lungs, along with 2,500 pancreas [3].

As per estimates, Spain reports the highest organ donation rate at 49.6 donors per million population, whereas India's donation rate stands at 0.26 per million population [4,5]. In order to meet the high demand, there is a need to raise the organ donation rate in India to 62 per million population, which constitutes a significant challenge [6]. Therefore, awareness generation regarding organ donation and motivating the country’s population has become imperative. Medical professionals, including doctors and nursing staff, play a crucial role in this aspect since they are directly involved in the treatment and care of patients. Medical and nursing students, being future professionals, also have a pivotal role to play. Their correct knowledge and positive attitude toward organ donation can lead to improved awareness and motivation among patients and their relatives.

Despite the significance of this topic, there is a dearth of studies in India on awareness regarding organ donation among medical professionals, particularly in the state of Bihar. Therefore, the present study was planned to assess the knowledge and attitude toward organ donation among students and professionals in medical colleges of Patna, Bihar.

## Materials and methods

Study design, setting, and study population

A cross-sectional study was conducted over six months from August 27, 2023, to March 31, 2024, in three government medical colleges in Patna. The study population included faculty, resident doctors, nursing staff, medical and nursing students, and interns from the three colleges.

Sample size and sampling procedure

The sample size was calculated based on the study finding of Shrivastav et al. conducted in a medical college in Gujarat, India, which reported 46.0% willingness for organ donation among medical undergraduates [7]. Considering an expected proportion of willingness as 46.0%, a confidence level of 95%, an absolute precision of 5%, a 10% non-response rate, and a design effect of 1.5, a sample size of 637 was estimated using the formula for estimation of a single proportion on Statulator web-based software (https://statulator.com/). Permission for data collection was obtained from the concerned authority of the selected medical colleges. Furthermore, information regarding the visit for data collection was communicated one day prior to the college authorities. Consecutive sampling was employed for the enrolment of study participants and written informed consent information was obtained from study participants. In total, 666 participants were included in the study.

Research instrument and data collection procedure

A pre-designed, structured, and validated questionnaire was developed after a thorough literature review to capture relevant data [8-13]. The questionnaire comprised three main sections, namely, (a) socio-demographic profile of participants, (b) knowledge regarding organ donation comprising 28 questions with binary responses (Yes/No) with each correct response earning one point, and (c) attitude toward organ donation, comprising of 12 questions with binary responses (Yes/No) with each correct response earning one point. A participant with a score equal to or above the median in the respective domain was classified as having “Good” knowledge and a “Positive” attitude regarding organ donation. Data were collected after obtaining written informed consent from study participants.

Data analysis

Data were collected from 666 study participants on Google Forms and transferred to Microsoft Excel for preliminary cleaning and coding. Data analysis was done using Jamovi software version 2.3.28 (available at https://www.jamovi.org/download.html). Descriptive statistics, such as mean, median, and standard deviation, were used for expressing quantitative variables like age, knowledge score, and attitude score while percentages and proportions were used for categorical variables like gender, religion, occupation, monthly income, and various components of the knowledge and attitude sections of the questionnaire. A chi-square test of association and linear regression analysis was employed to identify factors associated with the knowledge and attitude of study participants. A p-value less than 0.05 was considered statistically significant at a 95% confidence interval. The normality of plots and a plot of studentized residuals against predicted values. Homoscedasticity was checked through visual inspection.

Ethical considerations

The study was conducted after obtaining Institutional Ethics Committee (IEC) clearance (Reference Number: RDIAMS/Pat/2023/RAC/95). Emphasis was placed on voluntary participation, and study participants were allotted unique identifiers to ensure their anonymity.

## Results

Demographical characteristics of participants

The mean age of study participants was 23±4.24 (SD) (Table 1). The majority (504, 75.7%) of participants were in the age group of 21-30 years and were females (372, 55.9%). Furthermore, most participants were unmarried (600, 90.1%) and belonged to nuclear families (445, 66.8%). Maximum participation was from medical students and interns (349, 52.4%), followed by nursing students and staff (164,24.6%) and junior residents (126, 18.9%). Less participation was observed for senior residents (6.5%). A total of 186 (27.9%) participants had monthly earnings ranging from INR 50,000 to 10,00,000, while 179 (26.9%) had earnings ranging from INR 1,00,000 to 5,00,000. Across all groups of participants, good knowledge regarding organs that can be donated was observed for kidney, liver, heart, bone marrow, and cornea. Notably, there was a low level of knowledge regarding the donation of organs such as pancreas, lungs, and intestine.

**Table 1:** Demographic characteristics of participants (N=666)

Variable	Categories	Frequency (n)	Percentages (%)
Mean age of participants = 23±4.24			
Age (in years)	<20	108	16.2
	20-30	504	75.7
	>30	54	8.1
Gender	Female	372	55.9
	Male	294	44.1
Marital status	Married	66	9.9
	Unmarried	600	90.1
Type of family	Joint	221	33.2
	Nuclear	445	66.8
Education	Undergraduate and below	536	80.5
	Postgraduate and above	130	19.5
Occupation	Junior residents	126	18.9
	Medical students and interns	349	52.4
	Nursing students and staff	164	24.6
	Senior residents and consultant	27	4.1
Monthly income (in INR)	>10,00,000	43	6.5
	5,00,000-10,00,000	64	9.6
	1,00,000-5,00,000	179	26.9
	50,000-1,00,000	186	27.9
	25,000-50,000	107	16.1
	<25,000	87	13.1

Knowledge of participants regarding organ donation

Almost all participants across all groups have heard about organ donation and transplantation (Table 2). A significant percentage of participants (506, 76.0%) believe that organ donation has become a safe and easy procedure today. However, there is a difference in perception across groups, with consultants and junior/senior residents having a higher belief (128, 83.7%) in safety in the organ donation process that includes surgical procedures during organ transplantation compared to medical students/interns (249, 71.3%) and nursing students/staff (129, 78.7%). The study found that nursing students and staff have a higher knowledge (51.2%) about age eligibility for organ donation compared to medical students and interns (41.2%) and resident doctors and faculty (36.6%). This increased awareness may be due to the greater clinical exposure and interaction with patients that nursing students and staff experience compared to the other groups. A notable percentage of participants across all groups know organ donor cards. Consultants and residents group (62, 40.5%) along with nursing students/staff (74, 45.1%) have a higher awareness about donor cards compared to medical students/interns (90, 25.8%). Regarding the legal entity of a donor card, there is a difference in perception, with medical students/interns (256, 73.4%) having a higher belief that a donor card carries a legal entity than consultants and nursing students/staff (88, 57.5%). The majority of participants across all groups believed that organ or tissue donation does not disfigure the body (539, 80.9%). A significant percentage of participants (453, 68%) believed that people have to wait long for a donor transplant, with consultants having the highest percentage of positive responses.

**Table 2:** Knowledge of study participants regarding organ donation (N=666) *Chi-square test of association

Knowledge question (response)	Resident doctor and consultant (n1=153) n (%)	Medical students and interns (n2=349) n (%)	Nursing students and staff (n3=164) n (%)	Total (N=666) N (%)	P-value*
Have you heard about organ donation? (Yes)	153 (100)	348 (99.7 )	164(100)	666 (99.8 )	0.635
Have you heard about organ transplantation? (Yes)	152 (99.3 )	347 (99.4 )	161 (98.2 )	660 (99.1)	0.349
Organ donation has become a safe and easy procedure today (True)	128 (83.7 )	249 (71.3)	129 (78.7 )	506 (76.0 )	0.008
Is the demand for organ donation high compared to the present level of donation? (Yes)	142 (92.8)	305 (87.4 )	143 (87.2 )	590 (88.6 )	0.173
Is any age eligible for organ donation? (Yes)	56 (36.6 )	143 (41.0 )	84 (51.2 )	283 (42.5 )	0.022
Do you know what a donor card is? (Yes)	62 (40.5 )	90 (25.8 )	74 (45.1 )	226 (33.9 )	0.001
Does the donor card carry any legal entity? (No)	88 (57.5 )	256 (73.4 )	91 (55.5 )	435 (65.3 )	0.001
Does the donation of organs or tissue disfigure the body? (No)	112 (73.2 )	301 (86.2 )	126 (76.8 )	539 (80.9 )	0.001
Can organ transplantation be performed in any government or private hospitals? (No)	106 (69.3)	220 (63.0)	74 (45.1 )	400 (60.1 )	0.001
Do people have to wait for long for a donor transplant? (Yes)	121 (79.1 )	229 (65.6 )	103 (62.8)	453 (68.0 )	0.003

**Figure 1:**
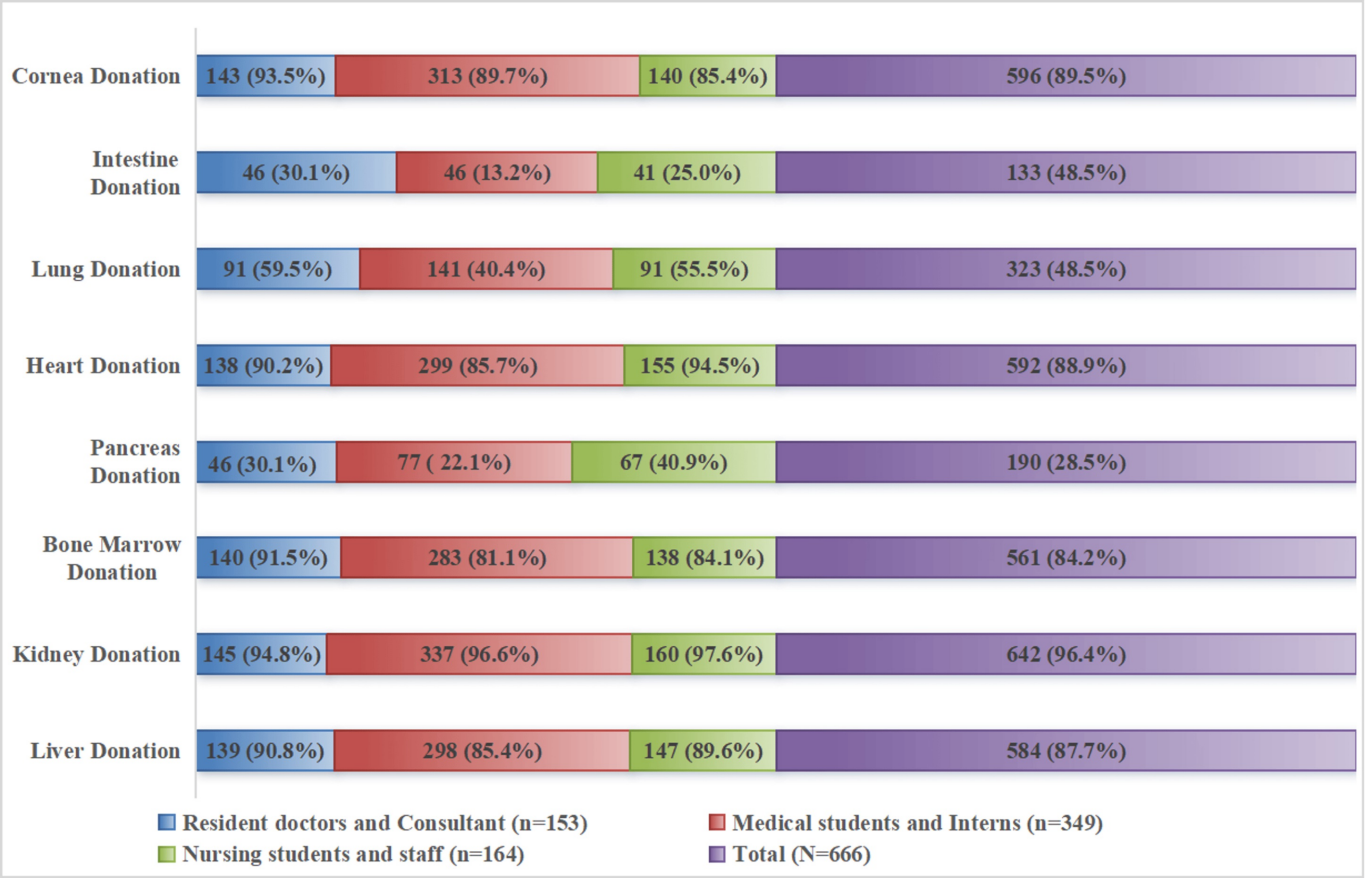
Distribution of participants with good knowledge regarding each organ(s), which is available for organ donation (N=666)

Across all groups of participants, a high level of awareness for the organ(s) available for donation was observed for kidney, liver, heart, bone marrow, and cornea. Notably, a low level of awareness observed among participants regarding organ(s) available for donation are the pancreas, lungs, and intestine (Figure 1).

Knowledge of participants related to legal and procedural components of organ donation

There is a significant difference in understanding between groups, with consultants/residents (75, 49.0%) showing a higher understanding of brain death being crucial for organ transplantation when compared to other groups (Table 3). The majority (597, 89.6%) of participants know that selling organs is illegal in India. However, there is a difference in understanding regarding the final decision of the donor regarding which organ to donate, with medical students/interns (149, 42.7%) having the highest understanding. Nursing students and staff have a higher understanding (103, 62.8%) that an immediate relative must consent when signing a donor card, compared to medical students and interns (154, 44.1%). This disparity may be due to medical students and interns spending more time in academic settings with less direct patient care experience, resulting in lower awareness of these legal requirements compared to nursing students and staff. There is a variation in understanding regarding the flexibility in changing the decision to donate organs, with consultants having the highest understanding that the decision can be changed. Most participants (482, 72.4%) understand that there will be no change in treatment once a potential donor signs the donor card. There is a higher understanding among consultants (133, 86.9%) regarding the time frame within which organ transplantation should be performed. The knowledge regarding donors can not ask for money for their organ donation is higher among consultants (114, 74.5%) while in other comparison groups; this understanding was poor.

**Table 3:** Knowledge of participants regarding legal entities related to organ donation (N=666) *Chi-square test of association

Knowledge questions on legal entities with positive responses	Resident doctor and consultant (n1=153) n(%)	Medical students and interns (n2=349) n (%)	Nursing students and staff (n3=164) n (%)	Total (N=666) N (%)	P-value*
To perform organ transplantation, brain death is the most important (Yes)	75 (49.0)	81 (23.2)	38 (23.2)	194 (29.1)	<0.001
Organ donation is not performed after cardiac death in India (Yes)	48 (31.4)	37 (10.6)	28 (17.1)	113 (17.0)	<0.001
Is it legal to sell organs in India? (No)	143 (93.5)	314 (90)	140 (85.4)	597 (89.6)	0.059
Is the decision of the donor final regarding which organ to donate? (No)	51 (33.3)	149 (42.7)	52 (31.7)	252 (37.8)	0.024
Does an immediate relative need to give consent while signing a donor card? (Yes)	74 (48.4)	154 (44.1 )	103 (62.8)	331 (49.7)	<0.001
Once a person decides to donate an organ, can his/her decision be changed? (No)	110 (71.9)	202 (57.9)	121 (73.8)	433 (65.0)	<0.001
Once a potential donor signs the donor card, is there any change in his/her treatment? (No)	120 (78.4)	258 (73.9)	104 (63.4)	482 (72.4)	<0.001
Organ transplantation should be performed within some time frame (True)	133 (86.9)	243 (69.6)	113 (68.9)	489 (73.4)	<0.001
Donors can't ask for money for their organ donation (True)	114 (74.5)	132 (37.8)	54 (32.9)	300 (45.0)	<0.001
Swap donation' can be done among families where organ donation is not possible because of a mismatch of blood groups (Yes)	60 (39.2)	110 (31.5)	71 (43.3)	241 (36.2)	0.024

**Figure 2:**
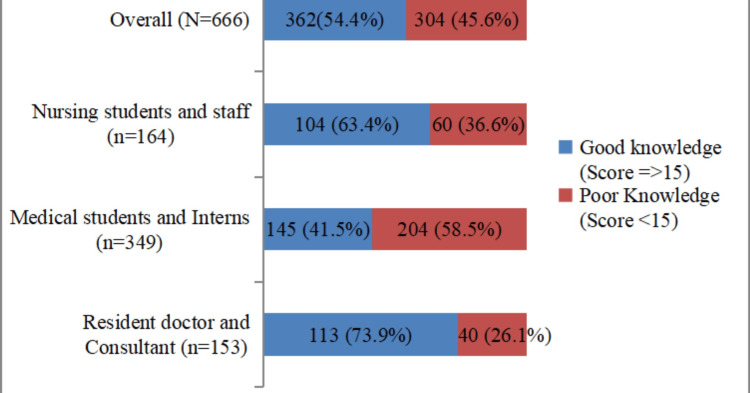
Distribution of participants according to knowledge regarding organ donation (N=666)

The overall median knowledge score obtained for participants for organ donation was 15. Participants who scored 15 or more were considered to have good knowledge. Approximately, 362 (54.4%) of participants exhibited a commendable understanding of various aspects of organ donation. Notably, resident doctors and consultants displayed the highest level of knowledge (113, 73.9%), whereas medical students and interns showed a comparatively poor understanding of organ donation (145, 41.5%) (Figure 2).

**Figure 3:**
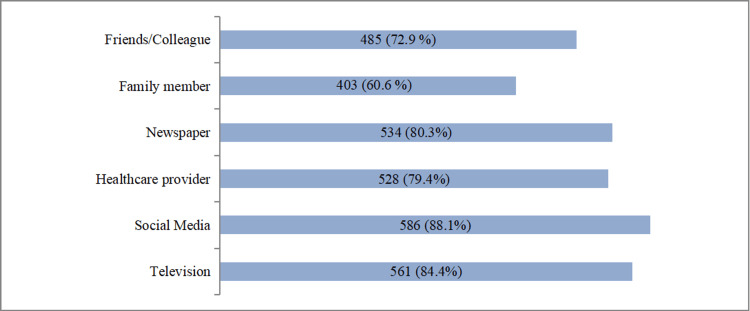
Source of information regarding organ donation among participants (N=666)

The study identified social media (586, 88.1%), television (561, 84.2%), and newspapers (534, 80.3%) as the most common sources of information regarding organ donation among participants (Figure 3).

Attitude of participants toward organ donation

Strong support was observed for organ donation and related campaigns across all groups of participants. Overall 614 (92.2%) of total participants support organ donation campaigns (Table 4). A significant percentage of participants (463, 69.5%) believed that their religion supports organ donation. A considerable proportion (438, 65.8%) did not believe organs are sold and bought in the black market. Most participants (553, 83%) believe organ donation is safe for the donor and effective for the recipient. A majority of participants (477, 71.6%) expressed their disagreement toward accepting money if they ever donate organs. There is a varying level of willingness for live donation, post-death organ donation, and body donation for academic/research purposes across various groups. Almost less than half of the respondents (279, 41.9%) expressed willingness to engage in live organ donation if needed with maximum support for live organ donation obtained from nursing students and staff. A considerable portion (479, 71.9%) of respondents were open to accepting an organ transplant if they ever needed one. A majority (413, 62%) of participants were willing for posthumous organ donation, which shows their desirability for organ donation. A smaller but still notable percentage (219, 32.9%) were willing to donate their bodies for academic or research purposes after death. There is a high willingness (570, 85.6%) to raise community awareness regarding organ donation. A relatively low percentage of participants (75, 11.3%) possess an organ donor card.

**Table 4:** Attitude of participants toward organ donation (N=666) *Chi-square test of association

Questions to check the attitude of participants toward organ donation	Resident doctors and consultant (n1=153) n (%)	Medical students and interns (n2=349) n (%)	Nursing students and staff (n3=164) n%	Total (N=666) N (%)	P-value*
Do you support organ donation and various campaigns related to it? (Yes)	144 (94.1)	312 (89.4%)	158 (96.3%)	614 (92.2%)	0.014
Do you think your religion supports organ donation? (Yes)	104 (68.0)	232 (33.5)	127 (77.4)	463 (69.5)	0.038
Do you think organs are sold and bought in the black market? (No)	49 (32)	117 (66.5)	127 (77.4)	438 (65.8)	0.003
Do you believe organ donation is safe for the donor? (Yes)	134 (87.6 )	216 (61.9)	127 (77.4)	477 (71.6)	< 0.001
Do you consider organ transplantation to be effective for the recipient? (Yes)	136 (88.9)	274 (78.5)	143 (87.2 )	553 (83.0 )	0.004
Would you like to accept money if you ever donate organ (s)? (No)	129 (84.3)	250 (71.6)	98 (59.8 )	477 (71.6)	<0.001
Are you willing to do a live donation (like a kidney) if needed? (Yes)	73 (47.7)	117 (33.5)	89 (54.3 )	279 (41.9)	<0.001
Will you accept an organ transplant for yourself, if ever the need arises? (Yes)	108 (70.6)	240 (68.8)	131 (79.9 )	479 (71.9 )	0.03
Are you willing to donate organ (s) after death? (Yes)	102 (66.7)	187 (53.6)	124 (75.6 )	413 (62.0 )	0.001
Are you willing to donate your body after death for academic/research purposes? (Yes)	39 (25.5)	103 (29.5)	77 (47.0 )	219 (32.9 )	0.001
Are you willing to raise community awareness regarding organ donation? (Yes)	140 (91.5)	283 (81.1)	147 (89.6)	570 (85.6 )	0.002
Do you possess an organ donor card? (Yes)	11 (7.2)	29 (8.3)	35 (21.3 )	75 (11.3 )	0.001

**Figure 4:**
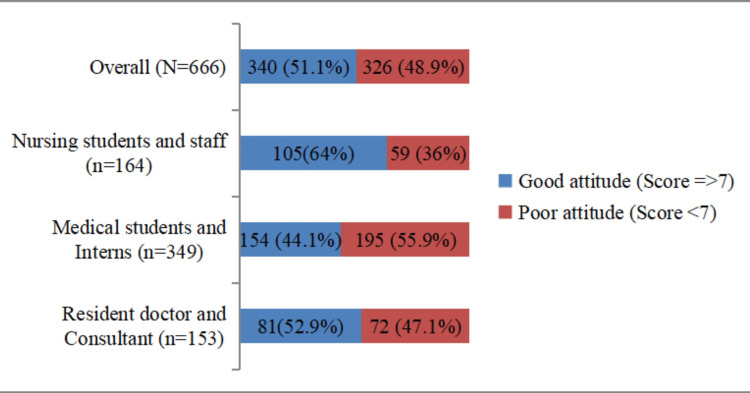
Distribution of participants according to attitude toward organ donation (N=666)

The overall attitudes were assessed by aggregating all responses in the attitude section of the questionnaire. The overall median attitude score of participants toward organ donation was seven. Participants who scored seven or more were considered to have good attitude. It was noted that 340 (51.1%) of participants had positive attitudes toward organ donation (Figure 4). Moreover, a higher proportion of nursing students and staff exhibited positive attitudes toward organ donation compared to residents and consultants and medical students and interns groups.

Multiple linear regression analysis to identify factors influencing attitude scores regarding organ donation

The multiple linear regression (MLR) analysis identified key predictors of attitude scores of participants toward organ donation. In univariate analysis, older age, male gender, Hindu religion, nuclear family structure, nursing profession, lower income, and awareness of organ donation were linked to more positive attitude scores. In multivariate analysis, religion, family type, occupation, and income level remained significant predictors, highlighting the influence of religious beliefs, family dynamics, profession, and socioeconomic status on attitudes toward organ donation (Table 5).

**Table 5:** Predictors of attitude score of participants regarding organ donation (N=666) *P-value ≤0.2 considered significant for univariate linear regression analysis **P-value <0.05 considered significant for multivariate linear regression analysis

Predictor		Univariate analysis		MLR analysis			
		Unstandardized coefficient estimate (B)	P-value*	Unstandardized coefficient estimate (B)	Confidence interval		P-value**
					Lower	Upper	
Age (in years)		0.0444	0.037	-0.0042	-0.0534	0.045	0.867
Gender	Male	-0.482	0.009	0.02871	-0.3396	0.397	0.878
	Female	(Ref)		(Ref)			
Religion	Other	-1.22	<0.001	-0.9673	-1.4357	-0.4989	<0.001
	Hindu	(Ref)		(Ref)			
Type of family	Nuclear	0.542	0.005	0.48384	0.1394	0.8283	0.006
	Joint	(Ref)		(Ref)			
Education	Undergraduate or below	-0.010	0.965				
	Post-graduate or above	(Ref)					
Occupation	Nursing students and staff	1.07	<0.001	0.59272	0.1461	1.0393	0.009
	Medical students/Interns/resident doctor/consultant	(Ref)		(Ref)			
Monthly income of the family (in INR)	>50,000	-0.465	0.021	-0.3819	-0.7481	-0.0158	0.041
	<50,000	(Ref)		(Ref)			
Marital status	Unmarried	-0.407	0.183	-0.4171	-1.1008	0.2665	0.231
	Married	(Ref)		(Ref)			
Knowledge score of participants regarding organ donation		0.26	<0.001	0.24521	0.2044	0.286	<0.001

## Discussion

Our study found that more than half of the participants understood organ donation well, but there were noticeable differences in knowledge levels across different groups. Specifically, residents and consultants showed the highest levels of knowledge, followed by nursing students/staff and medical students/interns. This trend is consistent with the findings of Reena et al., who also observed higher knowledge levels among medical undergraduates and interns compared to nursing students [14]. The above findings also indicate that as the level of medical education and clinical exposure increases, the understanding of organ donation increases [14,15].

Regarding awareness levels, it is encouraging to note that most participants in our study reported having heard of organ donation and transplantation. This finding is consistent with studies conducted among medical undergraduates by Ganta et al. and Swamy et al. [16,17]. However, despite this general awareness, there remains a low level of specific knowledge about organ donation among medical students. This gap could be attributed to two main factors: first, organ transplantation is not performed at all medical colleges in India, and second, this topic is not taught in depth in the medical curriculum across these institutions.

When examining knowledge about which organs can be donated, our study found high awareness levels among participants for kidney (96.4%), cornea (89.5%), liver (87.7%), and heart (88.9%) donation, but lower awareness for pancreas (28.5%), lung (48.5%), and intestine (20%). Similar findings have been noted in studies conducted in various medical colleges across India [16,18,19]. In contrast, a study in Telangana by Priyank et al. revealed a relatively low overall awareness level (37%) among undergraduates regarding identifying organs that can be donated [20]. Additionally, a study by Giri et al. in Maharashtra found that only half (46.94%) of medical students could identify organs to be donated, indicating regional variations in awareness levels [21]. This highlights the need for targeted educational interventions to enhance understanding of organ donation among medical professionals, particularly for lesser-known organs. 

In our study, approximately 34% of participants admitted to their knowledge about donor cards. Other studies conducted among medical students in India also demonstrated low awareness regarding donor cards [17,20]. There is a substantial need for increased awareness initiatives regarding organ donation registration-related activities among medical professionals and students in India. 

In our study, two-thirds of healthcare professionals admitted they would not accept money if they were ever making organ donations in the future. However, in another study by Priyanka et al., half of the medical undergraduates admitted that accepting money for organ donation is illegal [20]. In contrast, a study observed that 20.5% of medical undergraduates had misconceptions that payments are made to a person who donates organs [16]. These study findings suggest the need for more robust educational programs and awareness initiatives to address knowledge gaps and misconceptions about organ donation, ensuring that medical students and professionals are well-informed and ethically aligned with legal standards. 

Overall there was low awareness (29%) among participants regarding the fact brain death is the mandatory criterion for conducting organ donation while for residents and consultants, it was noted as high (49%) in our study. This could be attributed to the fact that in India, only qualified doctors, residents, and consultants are authorized to declare brain death because they possess the knowledge and skills necessary to ensure that the criteria for organ transplantation are met safely and ethically while the role of nurses is supportive during this process. This finding is further supported by other studies, which have observed similar findings [5-9,14-24]. A study by Sam et al. conducted among final-year medical students found that a large chunk of medical students (56.6%) were aware that the organ donation act in India recognizes brain death patients as eligible for organ donation [25]. This suggests that enhancing medical education and training regarding brain death and organ donation criteria across various groups of medical professionals could lead to more favorable outcomes.

In our study, 76% of healthcare professionals believed organ donation is a safe and effective procedure. Ganta et al. reported that 63.2% of respondents felt there were no complications associated with organ donation [16]. This highlights a significant level of confidence among healthcare professionals in the safety and efficacy of organ donation, which is critical for promoting positive attitudes and practices toward organ donation.

In our study, around 50% of healthcare professionals correctly answered that an immediate relative needs to give consent while signing a donor card. Similarly, a study in Maharashtra revealed that three-fifths of medical undergraduates were aware of whose consent is required for living and cadaveric donation [21]. A comparatively low proportion of medical students were aware of it in a study conducted in Kerala state [26]. Another study by Balwani et al. conducted among the general population observed that about two-fifths of participants believed that there is a requirement for the consent of the family before organ donation [27]. Thus, this is the consistent observation that while doctors possess a solid general understanding of organ donation, there remains a notable gap in their knowledge concerning the procedural and legal requirements associated with the organ donation process.

In our study, 80.9% of participants believed that organ donation does not cause any type of organ or tissue disfigurement. Priyanka et al. found that 29.6% of their respondents believed that organ donation causes body disfigurement [20]. Similarly, our study observed that 42.5% of healthcare professionals were aware of the age eligibility criteria for organ donation. Priyanka et al. found that 65.9% of participants were aware that people of any age are eligible for organ donation [20]. These results underscore the pressing need for an evolved educational framework. It is essential to prioritize the education of medical professionals through comprehensive training programs before reaching out to the general public. Such initiatives will foster a positive environment both within healthcare settings and in the broader community, ultimately promoting a better understanding of organ donation and thus, increasing the organ donation rate in India. 

One of the notable observations from our study is the positive attitude exhibited by a majority of healthcare professionals toward organ donation, with a significant percentage agreeing that religion does not oppose such acts. This aligns with the findings of Verma et al. where a considerable proportion of medical professionals demonstrated a favorable attitude toward organ donation across different regions [28].

However, our study also highlights variations in attitudes based on demographics, such as nursing staff and students showing a more favorable attitude compared to other groups. This finding is supported by Vincent et al., which noted a higher willingness among nursing students toward organ donation [8]. Conversely, Zirpe et al. reported differences between doctors and nurses regarding willingness to donate organs after death, indicating the need for further exploration into the factors influencing these variations [29]. Attitudes toward organ donation vary among different demographic groups, necessitating further investigation into underlying factors.

A crucial aspect brought to light by Mahajan et al. and Sindhu A. is the influence of religion on attitudes toward organ donation [9,30]. While our study and other studies indicate a positive trend regarding religion's role, Sindhu et al. highlighted that religion negatively affects attitudes toward organ donation [16,28,30]. This complexity suggests that the relationship between religion and attitudes toward organ donation is multifaceted, exhibiting both positive and negative effects. These variations may be closely tied to local socio-cultural contexts and the effectiveness of awareness campaigns conducted by local governments or their own educational institutions.

The willingness to donate organs varies significantly across different studies, with our research indicating a willingness percentage of 62%, compared to 55% in Chandigarh and 44.3% in Kerala [9,26]. Additionally, a study by Bathija et al. revealing nearly identical willingness among residents and interns indicates a consistent trend among healthcare professionals at different stages of their careers [31]. These discrepancies may be attributed to socio-economic factors, as highlighted in our findings (Table 4), which suggest that individuals with higher incomes tend to exhibit greater awareness regarding organ donation. Additionally, other contributing factors may include variations in funding sources, differences in educational quality, and the inconsistent implementation of organ transplantation policies among medical institutions in India. Collectively, these factors could lead to varying levels of training, exposure, and awareness among healthcare professionals.

The study's primary strength lies in its comprehensive scope and large sample size, encompassing a diverse range of participants from three major government medical colleges in Patna, Bihar. This broad representation enhances the generalization of the findings. Additionally, the study employs a structured and self-designed questionnaire developed from a thorough literature review, ensuring relevant and comprehensive data collection. The methodological rigor is further supported by MLR analysis, which helps understand the complex interactions between different factors influencing attitudes toward organ donation.

However, the study has notable limitations. The cross-sectional design restricts its ability to establish causality, only identifying associations between variables. The self-reported data introduces the risk of social desirability bias, where participants may provide socially acceptable responses rather than their true beliefs. Potential non-response bias and recall bias could also affect the study's reliability. Furthermore, no efforts were made to compare the results based on socio-economic differences or the type of medical institutions, such as state versus central. This oversight may introduce a potential source of bias in the findings. Additionally, the limited exploration of legal and ethical aspects of organ donation and the non-consideration of specific cultural factors further constrain the study's comprehensiveness. 

## Conclusions

This study highlighted the significant disparity between the knowledge and attitudes toward organ donation among medical professionals and students in Patna, Bihar. Despite high awareness levels, with nearly all participants familiar with organ donation and transplantation, there are notable gaps in specific knowledge areas such as the legalities and procedural aspects of organ donation. The findings reveal that residents and consultants generally possess better knowledge than medical and nursing students. Additionally, the majority of participants expressed support for organ donation campaigns, yet misconceptions about the safety and ethics of organ donation persist. These insights underscore the need for targeted educational interventions to address these knowledge gaps and foster a more supportive environment for organ donation. 
